# Impact of human papillomavirus vaccination knowledge, health beliefs, and self-efficacy on vaccination intention in adolescent sons in Korea: a descriptive survey study

**DOI:** 10.7586/jkbns.25.001

**Published:** 2025-02-20

**Authors:** Jiyeon Bark, Haejin Kim, So Im Ryu

**Affiliations:** 1 Department of Nursing, Changwon National University, Changwon, Korea; 2 College of Nursing, Kangwon National University, Chuncheon, Korea

**Keywords:** Papillomavirus vaccines, Intention, Parents, Adolescent, Men

## Abstract

**Purpose:**

This study aimed to identify the actor and partner effects of human papillomavirus (HPV) vaccination knowledge, health beliefs, and self-efficacy on parental intentions to vaccinate adolescent sons against HPV.

**Methods:**

The participants were 191 couples who met the eligibility criteria. Data were collected from June 2024 through internet communities in Gyeongsangnam Province. The actor-partner interdependence model was used to analyze the parent's actor and partner effects of HPV vaccination knowledge, health beliefs, and self-efficacy on parents' intentions to vaccinate their adolescent sons against HPV.

**Results:**

The actor effects of fathers' intentions to vaccinate their adolescent sons against HPV were perceived benefits and self-efficacy; however, the partner effect was not significant. The actor effects of mothers' intentions to vaccinate their adolescent sons against HPV were perceived susceptibility, perceived severity, perceived benefits, and self-efficacy, and the partner effects were fathers' perceived severity and benefits.

**Conclusion:**

This study indicates that fathers' intentions to vaccinate their adolescent sons against HPV may play an essential role in increasing HPV vaccination rates among adolescent sons.

## INTRODUCTION

Human papillomavirus (HPV) is a common sexually transmitted infection that affects two in five adults aged 15–59 years, and 75% of sexually active adults are infected [1]. HPV is the leading cause of cervical cancer, and efforts are underway worldwide to reduce the prevalence of HPV infection in women, including HPV screening, vaccination, and outreach programs [2,3]. HPV poses a serious threat to both men's and women's health. Oropharyngeal, oral, and laryngeal cancers caused by HPV are more common in men than in women [2], and HPV infection is the leading cause of male infertility [4].

The top seven countries in the Organization for Economic Cooperation and Development's Gross Domestic Product have recently actively implemented national HPV vaccination programs for men to increase their HPV vaccination rates and awareness [5]. It has been reported that vaccination of both men and women with the HPV vaccine can increase the population's overall herd immunity by 20%–30% compared to vaccinating women alone [6], making HPV vaccination of men a potentially powerful tool in the fight against cervical cancer [7]. However, despite the benefits of HPV vaccination in men, HPV vaccination rates among men in South Korea are very low (8.7%) [8]. This is believed to be owing to a combination of factors, including policies that limit the eligibility of domestic HPV vaccination programs to women [9], misconceptions that the HPV vaccine is a "cervical cancer vaccination" [10], and low knowledge among men [11-13].

The U.S. Centers for Disease Control and Prevention (CDC) recommends that adolescents, regardless of sex, receive the HPV vaccine at the age of 11 or 12 years before they become sexually active [1]. HPV National Vaccination Support Project in South Korea targets girls aged 12–17 years, but the government is considering expanding the program to include both boys and girls [14]. This is cost-effective from immunological and socioeconomic perspectives, given the earlier age of sexual initiation among adolescents [1,15,16]. International studies, including those from the U.S., have shown that HPV vaccination rates among male adolescents range from 12% to 45% [12,17]. Further, in Australia, a leader in adolescent HPV vaccination, the HPV vaccination completion rate among 15-year-old males in 2020 was 78%, which is very high considering that 80% of 15-year-old females were vaccinated [18]. Conversely, Korean studies have shown that HPV vaccination rates among boys aged 11–13 years are 0%–3.1%, significantly lower than the international rates [19,20]. Therefore, nursing assessments and active measures are needed to reassess HPV vaccination rates among Korean boys.

HPV vaccination intention is an individual's commitment to HPV vaccination and is a key antecedent of HPV vaccination behavior [21]. Studies based on the Health Belief Model (HBM) have been conducted to predict HPV vaccination intention [22-24]. Higher parental knowledge about HPV and HPV vaccine [23,25-27], health beliefs [13,24,28], and self-efficacy [20,23,25] have been associated with higher intentions to vaccinate children against HPV. Therefore, we sought to identify the factors that affect parents' intentions to vaccinate their adolescent sons against HPV based on the HBM.

Meanwhile, parents regularly communicate about their children's health and medical decisions, which reciprocally affects HPV vaccination intentions [29-31]. Previous research on children's HPV vaccination intentions has focused on mothers, making it appear that mothers' intentions are stronger [12,19,20]. Nonetheless, in reality, couples make decisions with input from both partners [29,31], and fathers are more likely to be receptive to their partners' input, especially if they have sons [30,31]. Few studies have been conducted on the parental interacting effects on the decision to vaccinate their sons against HPV; therefore, studies separating fathers and mothers to explore their different perspectives are needed [10,29].

The actor-partner interdependence model (APIM) is a statistical model that examines the interaction effects of distinguishable family members, such as married couples [32]. Lee et al. [29] emphasized that adolescent HPV vaccination requires an approach that considers the relational context rather than an individual-level approach; therefore, it is necessary to identify the interrelationship between parents and their adolescent sons' HPV vaccinations based on APIM.

Therefore, this examined parents' knowledge, health beliefs, and self-efficacy regarding parents' intentions to vaccinate their adolescent sons against HPV based on the HBM, using the APIM, we assessed whether an interaction effect influenced parents' intentions to vaccinate their adolescent sons against HPV. Which means contribute to developing intervention programs to increase HPV awareness and vaccination rates among adolescent sons.

## METHODS

### 1. Study design

**Figure 1.**
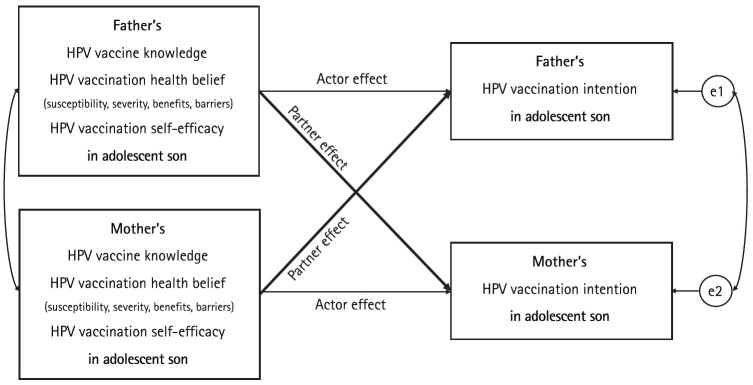
Framework of the study. e1 and e2: Measurement error variance. HPV = Human papillomavirus.

This descriptive survey aimed to determine the interactive effects of HPV vaccine knowledge, health beliefs, and self-efficacy on parents' intention to vaccinate their adolescent sons against HPV by applying APIM. Therefore, based on a review of the literature and related theories, a framework was constructed to determine parents' HPV vaccine knowledge, health beliefs, and self-efficacy as antecedent variables and their intention to vaccinate adolescent sons against HPV (Figure 1).

### 2. Participants

Parents with adolescent sons aged 11–17 years living in Changwon, Gyeongsangnam-do, South Korea, were included in the study as pairs. Parents with adolescent sons who had already received the HPV vaccine were excluded to identify the factors affecting HPV vaccination intentions for their adolescent sons. The size of the subject sample in a path analysis varies by researcher, but Hair et al. [33] suggest a minimum sample size of 100-150. Therefore, 150 pairs (300 participants) were initially selected for this study. To account for the 30% dropout rate reported in previous studies [19,20], the questionnaire was distributed to a total of 214 pairs (428 participants). After excluding four couples who did not respond, six couples that were not matched because only one parent completed the survey, seven couples in which the adolescent son was younger than 11 years, and six couples in which the parents switched surveys, a total of 191 couples (382 individuals) were included in the final analysis.

### 3. Instruments

#### 1) Participants' characteristics and HPV characteristics

General characteristics examined included parental age, number of children, son's age, parent's educational level, and monthly household income. The HPV characteristics included awareness of the mother's HPV vaccination status, heard about HPV vaccination, heard about men’s HPV infection, heard about men’s vaccination against HPV, recommended age for HPV vaccination, family discussion when planning their son's HPV vaccination, reasons for their son's lack of HPV vaccination, and who they would recommend to help them get their son vaccinated.

#### 2) Knowledge of HPV vaccine

This was measured using a scale developed by Kim and Ahn [34] and modified and supplemented by Park [35]. This study modified and supplemented the scale with appropriate items for parents with adolescent sons. Further, we used items with a Scale-Content Validity Index/Average (S-CVI/Ave) and Item Content Validity Index (I-CVI) of 1.0 after secondary content validation by one gynecologist, one pediatrician, and one professor of child nursing. The original number of items was 16. However, during the revision and refinement process, three items with redundant meanings were deleted based on the opinions of three experts, and 13 items were used. This was measured on a dichotomous scale, with 1 for correct answers, 0 for incorrect and don't know answers, and higher total scores indicating more significant knowledge of the HPV vaccine. At the time of scale development [34], the reliability was .87 by Kuder Richardson 20; in this study, the coefficient was .85 by Kuder Richardson 20.

#### 3) HPV vaccination-related health beliefs

This was developed by Choi et al. [36] based on the HBM and modified and supplemented by Park [21]. The scale is a 10-item, four-point Likert scale ranging from 1 for "strongly disagree" to 4 for "strongly agree," with higher total scores indicating stronger HPV vaccination-related health beliefs. The sub-items included perceived susceptibility (two items), perceived severity (two items), perceived benefits (two items), and perceived barriers (four items). Cronbach's α for Park's [21] instrument was .70 for perceived susceptibility, .75 for perceived severity, .84 for perceived benefits, and .73 for perceived barriers. Furthermore, Cronbach's α for this study was .66 for perceived susceptibility, .83 for perceived severity, .83 for perceived benefits, and .81 for perceived barriers.

#### 4) HPV vaccination-related self-efficacy

This was measured using the scale developed by Lipschitz et al. [37] and translated and modified by Lee et al. [38]. The scale is a 6-item, 5-point Likert scale ranging from 1, "I am not confident at all,"to 5, "I am very confident," with higher total scores indicating higher HPV vaccination self-efficacy. The original scale [37] had a Cronbach's α of .84, the Korean version [38] had a Cronbach's α of .85, and the present study had a Cronbach's α of .94.

#### 5) HPV vaccine intention

This tool was developed for adolescent parents by McRee et al. [39] and later modified and translated for women university students by Lee and Kim [40]. It was further adapted with items relevant to parents of adolescent sons. The revised primary survey items were validated for content by one gynecologist, one pediatrician, and one professor of pediatric nursing. Items with S-CVI/Ave and I-CVI of 1.0 were for use. The scale is a four-item, five-point Likert scale ranging from 1 (strongly disagree) to 5 (strongly agree), with higher total scores indicating higher HPV vaccination intention. Cronbach's α was .97 for the original tool [39], .81 for the Korean version [40], and .90 for this study.

All instruments were used after obtaining permission via email for its use and modification from the original and revised authors.

### 4. Data collection

The data collection period was between June 13, 2024, and June 30, 2024, and the participants were recruited by posting a recruitment notice through a local online community (https://www.bukmyeon.com) in Changwon, Gyeongsangnam-do. We collected the parents' phone numbers via a QR code on the recruitment post and texted each father and mother with a questionnaire so that they could take the mobile survey. The purpose and contents of the study were explained on the first screen of the mobile questionnaire, and if they understood the contents and agreed to participate, they were allowed to start the survey.

### 5. Data analysis

Data were analyzed using SPSS version 29.0 (IBM Corp., Armonk, NY, USA) and AMOS version 18.0 (IBM Corp., Armonk, NY, USA). Differences in general and HPV characteristics between parents were analyzed using real numbers, percentages, and χ^2^. Differences between parents' knowledge of the HPV vaccine, HPV vaccination health beliefs, HPV vaccination self-efficacy, and HPV vaccination intention were analyzed using a paired t-test. To determine the actor-partner effects of parental HPV vaccination knowledge, HPV vaccination-related health beliefs, and HPV vaccination-related self-efficacy on HPV vaccination intention, we conducted a path analysis to determine differences between the baseline model and the equivalence constraint model.

### 6. Ethical considerations

This study was approved by the Institutional Review Board of Changwon National University (7001066-202312-HR-068). The phone numbers collected for sending the online questionnaire and matching parent pairs were not used for research purposes and were immediately destroyed upon completion of data collection. If the participant interrupted the survey, all data was immediately deleted. The questionnaires were numbered and coded to ensure the anonymity and confidentiality of the participants. Participants were informed in the consent form that they would not be disadvantaged for not participating in the study. Additionally, all parents who participated received a mobile gift cards 5,000 Korean won each.

## RESULTS

### 1. Participants' general characteristics and HPV characteristics

The general characteristics of the study participants are presented in Table 1. Most participants were aged 40~44 years (55.5% and 60.7%, respectively), 83.8% had one child, and 29.3% had a son aged 12 years. Most parents had a college degree or higher, and 41.3% had a monthly family income of 5~6.99 million won. Among the HPV vaccine-related characteristics, fathers' and mothers' responses differed in several areas. These included awareness of their mothers' HPV vaccination status (χ^2^ = 27.52, *p* < .001), heard about HPV vaccine(χ^2^ = 23.02, *p* < . 001), awareness of men’s HPV infection (χ^2^ = 17.12, *p* < .001), awareness of whether men can be vaccinated against HPV (χ^2^ = 21.96, *p* < .001), and the recommended age for HPV vaccination (χ^2^ = 10.94, *p* < .001). Additionally, fathers and mothers were more likely to "involve both parents and sons" in the decision to vaccinate their sons. Fathers were more likely to cite a "lack of information," while mothers were more likely to cite concerns regarding "not safe" when asked why they did not vaccinate their sons. Both fathers and mothers were more likely to say healthcare providers would help them decide to vaccinate their sons.

### 2. Differences in parent's knowledge, health beliefs, self-efficacy, parents' intention to vaccinate their adolescent sons against HPV

Differences in HPV vaccination knowledge, health beliefs, self-efficacy, and intention to vaccinate sons against HPV infection among parents are shown in Table 2. HPV vaccine knowledge scores were significantly lower for fathers (8.67) than for mothers (9.08) (t = ╶2.67, *p* = .008). Health beliefs, self-efficacy, and intention to vaccinate their son against HPV were not significantly different between parents.

### 3. Actor–partner effects on HPV vaccination intention of parents

**Figure 2.**
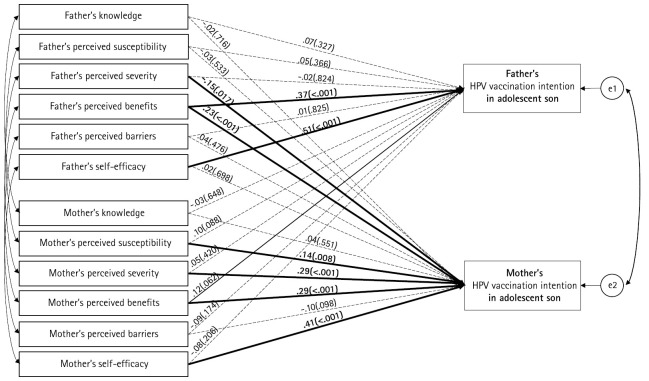
Model test : actor-partner effects on HPV vaccination intention of parents. e1 and e2: Measurement error variance. HPV = Human papillomavirus.

A path analysis was conducted to determine whether there was a dyadic interaction effect on parents' intention to vaccinate their sons against HPV. The results are shown in Table 3 and Figure 2. The actor effect on fathers' intentions to vaccinate their sons was significant for perceived benefits (β = .37, *p* < .001) and HPV vaccination self-efficacy (β = .51, *p* < .001), whereas the partner effect was not important. The actor effect on mothers' intention to vaccinate their sons for HPV was significant for perceived susceptibility (β = .14, *p* = .008), perceived severity (β = .29, *p* < .001), perceived benefits (β = .29, *p* < .001), and HPV vaccination self-efficacy (β = .41, *p* < .001). Partner effects were significant for fathers' perceived severity (β = ╶.15, *p* = .017) and perceived benefits (β = .23, *p* < .001).

For HPV vaccination-related perceived severity and perceived benefits, where the actor and partner effects were significant on mothers' intention to vaccinate their sons, an equivalence constraint model was set up to test for differences from the baseline model. A statistically significant χ^2^ difference was found for perceived severity (△χ^2^ = 17.14, *p* < .001), indicating that mothers' perceived severity was more influential than fathers' perceived severity on mothers' intention to vaccinate their sons. In contrast, mothers' perceived benefits were more effective than fathers' perceived benefits. Nonetheless, there was no significant χ2 difference (△χ^2^ = 0.36, *p* = .550).

## DISCUSSION

This study used the APIM to examine how parents' HPV vaccination knowledge, health beliefs, and self-efficacy affected their intentions to vaccinate their adolescent sons against HPV. Specifically, the study aimed to identify both the effects of a parent's own factors (actor effects) and the influence of their partner's factors (partner effects) on vaccination intentions.

We first examined the differences in HPV characteristics associate with awareness of HPV vaccine between parents. We found significant differences in awareness of the HPV vaccine, reasons for not being vaccinated, and knowledge of the HPV vaccine. Fathers were more likely to be unaware of whether mothers had been vaccinated against HPV and were less likely than mothers to have heard of the HPV vaccine. Furthermore, they were less likely to have heard of HPV infection in men, to be vaccinated against HPV in men, and to be aware of the recommended age for HPV vaccination. This is consistent with previous studies that examined fathers' intentions to vaccinate their children against HPV [10,12,13], which also found lower levels of awareness among fathers than among mothers. Currently, HPV vaccination is promoted by the CDC [41]. However, much of the communication is focused on "cervical cancer prevention," and the models and illustrations in videos and leaflets are predominantly women [41]. This suggests that information about HPV and the HPV vaccine is aimed at women [41]. Therefore, national communication strategies to raise awareness about HPV and HPV vaccines should be reoriented to engage men. In particular, it emphasizes that HPV can also affect men and that it is necessary to provide information about the risk of HPV infection and vaccination in men. This will require developing educational materials and campaigns tailored to men, including outreach efforts focused on raising awareness of the availability and effectiveness of HPV vaccination in men. I think it is important to adopt an approach that places the HPV vaccine in the broader context of men's health. For example, actively providing information that the HPV vaccine also plays a vital role in preventing several types of cancers in men, including oropharyngeal, oral, laryngeal, and anal cancers, may help men perceive HPV vaccination as an essential way to protect their health. For example, providing information about the HPV vaccine’s role in preventing various types of cancers in men is crucial. These include oropharyngeal, oral, laryngeal, and anal cancers. Such information may help men perceive HPV vaccination as an essential way to protect their health.

When we identified significant variables influencing parents' intentions to vaccinate their sons against HPV, Perceived benefits and self-efficacy were significant for fathers, and perceived susceptibility, severity, benefits, and self-efficacy were significant for mothers. These findings are consistent with previous research showing that parents' HPV vaccination-related health beliefs [13,42,43] and self-efficacy [19,20,25] affects their children's HPV vaccination intentions. Meanwhile, the variables that were commonly significant for parents were perceived benefits and self-efficacy. This is consistent with previous studies [13,44] that suggest that parental health beliefs and self-efficacy are essential predictors of HPV vaccination intention in children. Yeom and Lim [45] highlighted the need for HPV-related educational programs targeting community parents to improve awareness of HPV vaccination and increase HPV vaccination rates among their children. Therefore, to increase parents' intentions to vaccinate their children against HPV, educational programs should be designed to enhance parents' perceived benefits and self-efficacy. The content of these programs should include reducing the burden of HPV vaccination and understanding the actual vaccination process.

We examined partner effects on parents' intentions to vaccinate their sons against HPV and found that fathers were not affected by mothers. However, mothers' intentions to vaccinate their sons against HPV were affected by fathers' perceived severity and benefits. No prior studies have analyzed parents' intentions to vaccinate their sons against HPV using parental interaction effect, making comparisons difficult. Nevertheless, we believe that the father's partner effect observed only for mothers may be due to the gender differences in parental vaccine hesitancy [46]. Mothers tend to be more hesitant than fathers when it comes to vaccinating their children [46]. Studies that have examined HPV vaccination intentions using a variable called subjective norms have shown that mothers are highly influenced by those around them when considering HPV vaccination for their children [19,20]. Therefore, in South Korea, where male HPV vaccination is not universally available, the influence of husbands may have played a role in mothers' hesitation to vaccinate their sons. These findings suggest the need for HPV vaccination education and campaigns that consider parental interactions, particularly programs that support fathers and mothers in respecting each other's input in decision-making and making collaborative decisions for their children's health. Finding ways to reconcile differences and build consensus among parents will be essential in successfully implementing HPV vaccination.

The above discussion confirms that fathers affect not only their own but also their mothers' intentions to vaccinate their sons against HPV. Therefore, awareness-raising programs that involve both parents and promotional strategies that actively engage fathers are needed to increase HPV vaccination rates among adolescent sons. These findings may contribute to the prevention of HPV-related diseases in adolescents. However, the generalizability of these results is limited because this was a convenience sample study that only included parents from a specific region of Gyeongsangnam-do. Nevertheless, this study is significant because it is the first to identify actor and partner effects on parents' intentions to vaccinate their sons against HPV. This study also confirmed the low awareness of HPV vaccination among men and their parents, which can be used as a rationale for ongoing national support for HPV vaccination in men. Altogether, these findings suggest that fathers' health beliefs influence mothers' decisions regarding their adolescent sons' health. Further research is needed to develop educational programs to increase awareness of HPV and HPV vaccines among parents of adolescent sons, particularly fathers.

## CONCLUSION

This study applied APIM to examine actor and partner effects on parents' intention to vaccinate their adolescent sons against HPV. The results showed that fathers' perceived benefits and self-efficacy related to their own HPV vaccination affected their HPV vaccination intentions for their adolescent sons. Mothers' perceived susceptibility, perceived severity, perceived benefits, and self-efficacy related to their own HPV vaccination affected their HPV intention to vaccinate their adolescent sons. It was affected by fathers' perceived severity and perceived benefits. Specifically, mothers' and fathers' HPV-related health beliefs affected mothers' intentions to vaccinate their adolescent sons against HPV. These findings provide an essential foundation for research on HPV vaccination among adolescent men.

## Figures and Tables

**Table 1.** Comparison of Parental General and HPV-related Characteristics between Fathers and Mothers

Characteristics	Categories	Fathers (n = 191)		Mothers (n = 191)		χ^2^	*p*
		n (%)		n (%)			
Age (yr)	≤ 39	14 (7.3)		42 (22.0)		28.36	< .001
	40~44	106 (55.5)		116 (60.7)			
	≥ 45	71 (37.2)		33 (17.3)			
Number of children	1	160 (83.8)					
	≤ 2	31 (16.2)					
Son's age (yr)	11	30 (15.7)					
	12	56 (29.3)					
	13	33 (17.3)					
	14	17 (8.9)					
	15	34 (17.8)					
	16	15 (7.9)					
	17	6 (3.1)					
Education level	< College	28 (14.7)		30 (15.7)		0.08	.776
	≥ College	163 (85.3)		161 (84.3)			
Monthly income (10,000 KRW)	< 500	45 (23.6)					
	500~699	79 (41.3)					
	≥700	67 (35.1)					
Awareness							
Mother's (wife's) HPV vaccination state	Yes	69 (36.1)		81 (42.4)		27.52	< .001
	No	83 (43.5)		104 (54.5)			
	Unknown	39 (20.4)		6 (3.1)			
Heard about HPV vaccination	Yes	129 (67.5)		168 (88.0)		23.02	< .001
	No	62 (32.5)		23 (12.0)			
Men's HPV infection	Yes	121 (63.4)		157 (82.2)		17.12	< .001
	No	70 (36.6)		34 (17.8)			
Men's HPV vaccination	Yes	100 (52.4)		144 (75.4)		21.96	< .001
	No	91 (47.6)		47 (24.6)			
Recommended age for HPV vaccination	Yes	66 (34.6)		98 (51.3)		10.94	< .001
	No	125 (65.4)		93 (48.7)			
Decision-making about HPV vaccination	Alone	1 (0.5)		1 (0.5)		0.57	.926^†^
	Partner	82 (42.9)		81 (42.4)			
	Son	22 (11.5)		19 (9.9)			
	Partner and son	86 (45.1)		90 (47.2)			
Reasons for not getting vaccinated	Lack of information	44 (23.0)		24 (12.6)		20.30	.005
	Not safe	42 (22.0)		56 (29.3)			
	No effect	8 (4.2)		16 (8.4)			
	Unknown recommended age	32 (16.8)		35 (18.3)			
	Lack of knowledge about HPV vaccination for boys	29 (15.2)		15 (7.9)			
	Not necessary for boys	9 (4.7)		8 (4.2)			
	Barriers to 3-dose schedule	8 (4.2)		4 (2.1)			
	Younger than the recommended age	19 (9.9)		33 (17.2)			
An important recommender	Healthcare provider	87 (45.5)		79 (41.4)		2.02	.733
	Teacher	20 (10.5)		26 (13.6)			
	Friend	18 (9.4)		17 (8.9)			
	Family	62 (32.5)		62 (32.4)			
	No one helpful	4 (2.1)		7 (3.7)			

HPV = Human papillomavirus. ^†^Fisher's exact test.

**Table 2.** Differences in Parents’ Knowledge, Health Belief, Self-efficacy, and HPV Vaccination Intention (N = 191 pairs)

Variables	Father (n = 191)				Mother (n = 191)				t	*p*
	M	SD	Skew	Kurtosis	M	SD	Skew	Kurtosis		
HPV vaccine knowledge	8.67	2.71	╶0.69	0.04	9.08	2.65	╶0.89	0.77	╶2.67	.008
HPV vaccination health beliefs										
Perceived susceptibility	3.98	1.41	0.42	╶0.32	4.04	1.36	0.44	╶0.29	╶0.60	.550
Perceived severity	5.86	1.70	╶0.59	╶0.64	5.99	1.61	╶0.72	╶0.35	╶1.28	.202
Perceived benefits	6.52	1.47	╶1.07	0.85	6.63	1.50	╶1.25	1.13	╶1.11	.269
Perceived barriers	10.08	2.97	0.00	╶0.71	9.96	2.81	╶0.12	╶0.63	0.69	.493
HPV vaccination self-efficacy	19.88	6.17	╶0.26	╶1.03	19.76	6.17	╶0.16	╶1.27	0.32	.748
HPV vaccination intention	15.56	3.27	╶1.39	1.63	15.70	3.37	╶1.41	1.56	╶0.72	.473

HPV = Human papillomavirus; M = Mean; SD = Standard deviation; Skew = Skewness.

**Table 3.** Results of the Equivalence Constraint Model (N = 191 pairs)

Variables	Fathers			Mothers			△χ2	*p*
	Path	β	*p*	Path	β	*p*		
Mother’s intention	← Perceived severity	╶.15	.017	← Perceived severity	0.29	< .001	17.14	< .001
	← Perceived benefits	0.23	< .001	← Perceived benefits	0.29	< .001	0.36	.550

△χ= χ2 difference.

## Data Availability

The data that support the findings of this study are available from the corresponding author upon reasonable request.
